# The Role of Glutamate Dehydrogenase (GDH) Testing Assay in the Diagnosis of *Clostridium difficile* Infections: A High Sensitive Screening Test and an Essential Step in the Proposed Laboratory Diagnosis Workflow for Developing Countries like China

**DOI:** 10.1371/journal.pone.0144604

**Published:** 2015-12-11

**Authors:** Jing-Wei Cheng, Meng Xiao, Timothy Kudinha, Zhi-Peng Xu, Lin-Ying Sun, Xin Hou, Li Zhang, Xin Fan, Fanrong Kong, Ying-Chun Xu

**Affiliations:** 1 Department of Clinical Laboratory, Peking Union Medical College Hospital, Chinese Academy of Medical Sciences, Beijing, China; 2 Graduate School, Peking Union Medical College, Chinese Academy of Medical Sciences, Beijing, China; 3 Centre for Infectious Diseases and Microbiology Laboratory Services, ICPMR–Pathology West, Westmead Hospital, University of Sydney, Darcy Road, Westmead, New South Wales, Australia; 4 The Charles Sturt University, Leeds Parade, Orange, New South Wales, Australia; 5 Teaching and Research Section of Clinical Laboratory, School of Public Health, Taishan Medical School, Taian, Shandong, China; Laurentian University, CANADA

## Abstract

The incidence and severity of *Clostridium difficile* infection (CDI) in North America and Europe has increased significantly since the 2000s. However, CDI is not widely recognized in China and other developing countries due to limited laboratory diagnostic capacity and low awareness. Most published studies on laboratory workflows for CDI diagnosis are from developed countries, and thus may not be suitable for most developing countries. Therefore, an alternative strategy for developing countries is needed. In this study, we evaluated the performance of the Glutamate Dehydrogenase (GDH) test and its associated workflow on 416 fecal specimens from suspected CDI cases. The assay exhibited excellent sensitivity (100.0%) and specificity (92.8%), compared to culture based method, and thus could be a good screening marker for *C*. *difficile* but not for indication of toxin production. The VIDAS CDAB assay, which can detect toxin A/B directly from fecal specimens, showed good specificity (99.7%) and positive predictive value (97.2%), but low sensitivity (45.0%) and negative predictive value (88.3%), compared with PCR-based toxin gene detection. Therefore, we propose a practical and efficient GDH test based workflow strategy for the laboratory diagnosis of CDI in developing countries like China. By applying this new workflow, the CDI laboratory diagnosis rate was notably improved in our center, yet the increasing cost was kept at a minimum level. Furthermore, to gain some insights into the genetic population structure of *C*. *difficile* isolates from our hospital, we performed MLST and PCR toxin gene typing.

## Introduction

*Clostridium difficile* is a Gram-positive, spore-forming, anaerobic rod, which is an important cause of antibiotic associated diarrhea. *C*. *difficile* infection (CDI) is a major nosocomial disease, with clinical symptoms ranging from asymptomatic colonization, diarrhea to toxic megacolon, bowel perforation, sepsis, shock and death [1]. Since the 2000s, there has been a significant increase in the incidence and severity of CDI in North America and Europe, placing a huge financial burden on health care systems worldwide [2]. Consequently, in 2013, CDI was considered an urgent public health threat by the U. S. Centers for Disease Control and Prevention [3]. However, due to low awareness, possibly emanating from limited laboratory diagnostic capacity, low sample submission rate, and lack of high-quality surveillance systems [4, 5], CDI is not widely recognized in Asia, including China. Most publications on workflow for the laboratory diagnosis of CDI are from Europe, and thus may not be suitable for developing countries with limited resources. As such, alternative workflow strategies are urgently needed to address this issue in developing countries.

CDI diagnosis is based on a combination of clinical history and laboratory detection of *C*. *difficile* toxin in the feces or cultured isolates [6, 7]. PCR-based commercial kits, which offer high sensitivity and specificity [8, 9], are widely used for CDI diagnosis in developed countries, but their costs exceed the affordability of many laboratories in developing countries. In China, PCR-based tests are not approved for use, and thus culture remains the main diagnostic method.

Glutamate dehydrogenase (GDH) is a constitutive enzyme produced in large amounts by all strains of *C*. *difficile* independent of toxigenicity. GDH is, therefore, easily detected in feces, which makes it a good screening marker for *C*. *difficile*. In order to improve the laboratory diagnostic capacity for CDI investigation, previous studies have recommended the use of *C*. *difficile* GDH as a preliminary screening test, followed by further confirmatory tests for toxin production and possession of toxin genes [10–13]. Due to low awareness of CDI, the GDH assay has not been approved for use in China, though indications are that this will happen before the end of 2015.

Moreover, there is limited data on the effectiveness of the assay in the diagnosis of CDI in developing countries. To address these concerns, we evaluated the performance of GDH assay in detecting *C*. *difficile* from fecal samples in a Chinese hospital clinical laboratory. And, based on a comprehensive consideration of the sensitivity, specificity, turnaround time and costs of different *C*. *difficile* testing assays, we proposed a practical workflow for future laboratory diagnosis of CDI in developing countries. Finally, to gain some insight into the genetic population structure of *C*. *difficile* isolates, we studied the MLST and PCR toxin gene types of our isolates.

## Materials and Methods

### Ethics

The study was approved by the Human Research Ethics Committee of Peking Union Medical College Hospital (No. S-263). Written informed consent was obtained from patients for the use of the samples in research.

### Sample collection and study design

Consecutive, non-repetitive fecal specimens (n = 416) were collected from patients presenting with diarrheal symptoms at the Peking Union Medical College Hospital, from August 2012 to July 2014. All the fecal specimens were stored at 4°C before testing, and were simultaneously tested by VIDAS *C*. *difficile* GDH assay (bioMérieux, Marcy l'Etoile, France), VIDAS *C*. *difficile* Toxin A&B (bioMérieux, Marcy l'Etoile, France), and culture-based method followed by molecular detection of toxin genes (see below for details).

### VIDAS *C*. *difficile* GDH and Toxin A&B (CDAB) detection

All the specimens were tested simultaneously for GDH and CDAB by commercial VIDAS kits (bioMérieux, Marcy l'Etoile, France), according to manufacturer’s instructions. Negative results for GDH assay were defined as optical density (OD) 450/630 nm < 0.10, and positive results were defined as OD450/630 nm ≥ 0.10. The fluorescence intensity for CDAB of < 0.13, ≥ 0.13 to < 0.37 and ≥ 0.37 were reported as negative, equivocal and positive, respectively.

### Sample culture and MALDI-TOF MS identification

All the specimens were cultured in appropriate media and identification of *C*. *difficile* was done according to the laboratory’s current routine laboratory workflows. Generally, the specimens were cultured on cycloserine-cefoxitin fructose agar (CCFA) in anaerobe condition at 35°C for 48 h. Suspect *C*. *difficile* colonies were tested by matrix-assisted laser desorption/ionization time-of-flight mass spectrometry (MALDI-TOF MS) with MALDI Biotyper version 3.1 (Bruker Daltonics GmbH, Bremen, Germany) according to the manufacturer’s instructions. Isolates with identification scores of ≥2.0 were considered to be accurately identified to species level.

### Toxin gene detection and multilocus sequence typing (MLST)

The genomic DNA of the *C*. *difficile* isolates was extracted by QIAamp DNA Mini Kit (QIAGEN, Hilden, Germany) following the manufacturer’s instructions. A 5-plex PCR was performed to detect 4 toxin genes, namely *tcdA*, *tcdB*, *cdtA* and *cdtB*, and the 16S rDNA of *C*. *difficile*, as previously described by Persson *et al*. [14]. MLST was performed by sequencing seven gene loci (*adk*, *atpA*, *dxr*, *glyA*, *recA*, *sodA* and *tpi*) as previously developed by Griffiths *et al*. [15]. The seven gene loci sequences were submitted to the PubMLST sequence query page (http://pubmlst.org/cdifficile/) to obtain the allele, clade and sequence types (ST).

### Statistical analysis

Statistical analyses were performed using SPSS software (version 17.0, IBM, New York, USA). The sensitivity, specificity, positive predictive value (PPV) and negative predictive value (NPV) were calculated.

## Results

### General clinical information

About 64% (264/416) of the patients were from Medical Department, 18.5% (77/416) from Outpatient and Emergency Departments, 7.2% (30/416) from Surgery Department, 5.0% (21/416) from ICU, and 5.8% (24/416) were from other Departments (Gynecology, Obstetrics and Pediatrics Departments). The average age of the 416 patients, which included 220 males and 196 female patients, was 43.8±21.3 years (ranged from 3–95).

### Performance of the VIDAS GDH assay for detection of *C*. *difficile* compared with routine culture

Among the 416 fecal specimens tested, 32.2% (134/416) were positive for GDH. In comparison to the GDH assay, 26.9% (112/416) of the specimens were *C*. *difficile* culture positive. About 5% (22 of 416) specimens were positive for GDH but negative for culture. Compared with routine *C*. *difficile* culture, the sensitivity, specificity, PPV and NPV of VIDAS GDH assay were 100.0%, 92.8%, 83.6% and 100.0%, respectively (Table 1).

**Table 1:** Comparison of the diagnostic methods for *C*. *difficile* infections in this study.

Testing methods	Percentage of (95% confidential interval)				TAT (h)	Costs (US$)
	Sensitivity	Specificity	PPV	NPV		
**GDH**	100 (96.8–100.0)	92.8 (89.3–95.4)	83.6 (76.2–89.4)	100 (98.7–100.0)	2	8
**Culture**	Reference method for GDH				48	15
**CDAB**	45 (33.9–56.5)	99.7 (98.6–99.9)	97.2 (85.8–99.9)	88.3 (84.7–91.4)	2	8
**Toxin gene detection**	Reference method for CDAB				50	20
**Proposed workflow**	Not applicable				15.1^a^	15.6^a^

Abbreviations: GDH, glutamate dehydrogenase; PPV, positive predictive value; NPV, negative predictive value; TAT, turnaround time.

^a^ The TAT and costs of the proposed workflow were calculated by average value of the 416 fecal specimens.

### Performance of VIDAS CDAB assay compared with molecular assay for toxin detection

Of the 416 fecal samples tested, 19.2% (80/416) were positive for toxin genes by PCR-based detection, and 36 (45.0%) of these samples were CDAB positive or equivocal. Furthermore, among the 336 PCR negative samples, one (0.3%) sample was equivocal for CDAB, and the rest negative. Compared with PCR-based toxin gene detection, the sensitivity, specificity, PPV and NPV of VIDAS CDAB were 45.0%, 99.7%, 97.2% and 88.3%, respectively (Table 1).

Amongst the 134 GDH positive fecal samples, 22.4% (30/134) and 9.0% (12/134) were positive and equivocal for VIDAS CDAB testing, respectively. All the GDH negative fecal samples (n = 282), were also negative for VIDAS CDAB assay.

### A proposed GDH-based diagnostic workflow for improved laboratory diagnosis of CDI in developing countries

**Fig 1:**
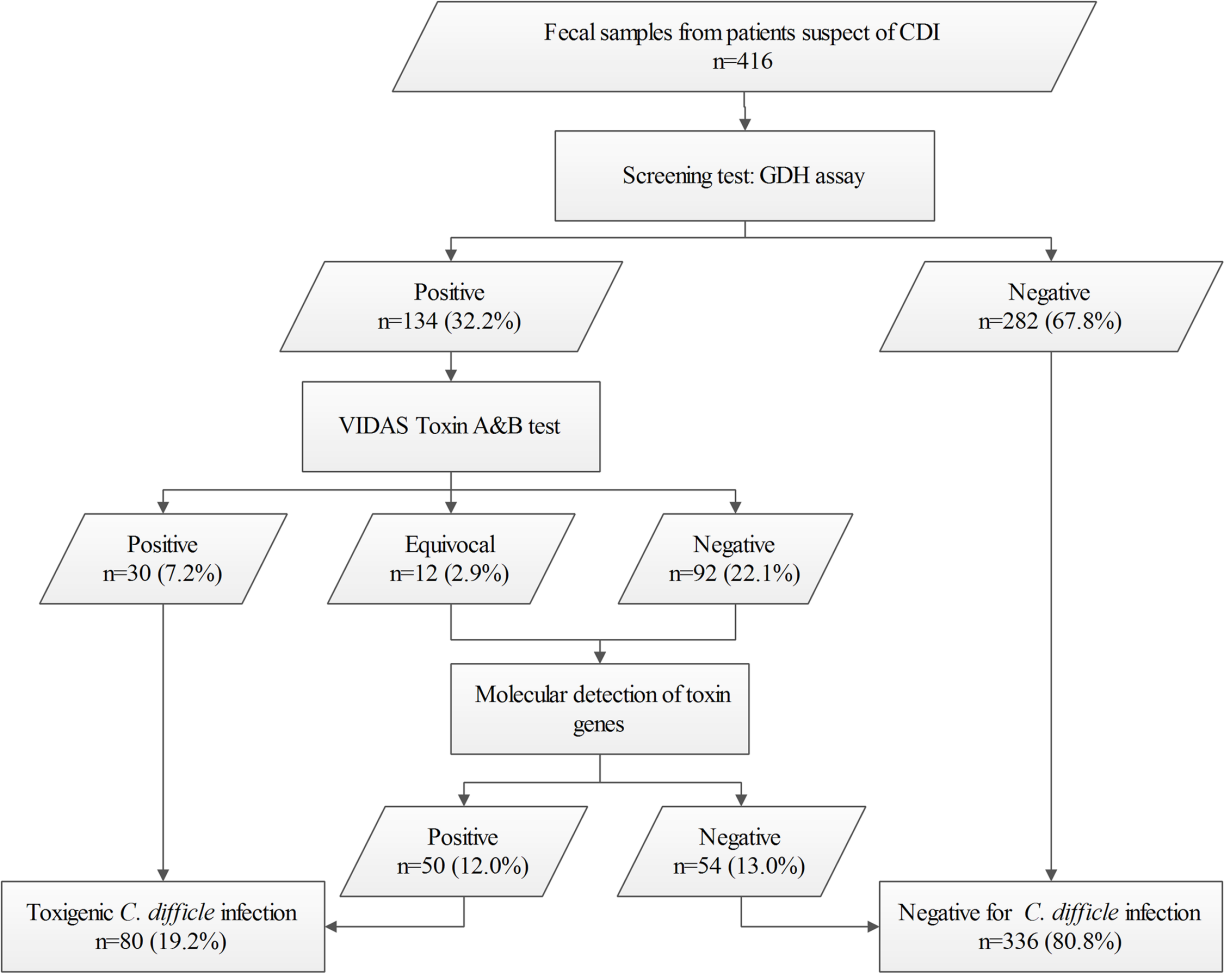
A proposed GDH-based workflow for laboratory diagnosis of *C*. *difficile* infections (CDI). Abbreviations in the figure: GDH, glutamate dehydrogenase. Cell cytotoxicity neutralization assay or toxigenic culture could be used as alternative testing methods for determining toxigenicity of *C*. *difficile* where molecular methods were not available.

To improve the laboratory diagnostic capacity for CDI in developing countries, we propose a practical and efficient workflow, based on a combination of testing methods in laboratory diagnosis of CDI (Fig 1). The first step is to screen the suspect CDI fecal specimens by GDH assay, with negative results directly reported to physicians. For GDH positive specimens, CDAB testing should be performed subsequently to detect toxin production. If the CDAB results are positive, laboratory diagnosis of CDI can be made. Samples with equivocal or negative CDAB results should be referred for further testing, such as molecular detection of toxin genes, toxigenic culture (TC) or cell cytotoxicity neutralization assay (CCNA). By applying this new workflow in our lab, the CDI diagnosis rate was notably improved from 8.2% (30/416) to 19.2% (80/416), whilst keeping the rising costs at a reasonable minimum (from US$8 to US$15.6) (Table 1).

### Toxin genotypes and MLST of the 112 *C*. *difficile* culture positive isolates

Of the *C*. *difficile* culture positive 112 isolates, 54 (48.2%) were *tcdA*-positive, *tcdB*-positive and *cdtA/cdtB*-negative (A^+^B^+^CDT^-^); 32 (28.6%) were *tcdA*-negative, *tcdB*-negative and *cdtA/cdtB*-negative (A^-^B^-^CDT^-^); and 20 (17.9%) were *tcdA*-negative, *tcdB*-positive and *cdtA/cdtB*-negative (A^−^B^+^CDT^−^). The remaining six (5.3%) isolates were *tcdA*-positive, *tcdB*-positive and *cdtA/cdtB*-positive (A^+^B^+^CDT^+^).

Using MLST, the 112 culture positive *C*. *difficile* isolates were classified into 23 STs, with the majority (68.8%) belonging to clade 1. ST-3 was the most common ST (18.8%, 21/112), followed by ST-54 (13.4%, 15/112) and ST-37 (9.8%, 11/112). The six A^+^B^+^CDT^+^ isolates were grouped into ST-1 and ST-5. In addation, all the 20 A^−^B^+^CDT^−^ isolates were ST-37 and ST-81 (Table 2). Generally, there were no obvious correlations between clonal clusters generated and the isolate hospital source departments.

**Table 2:** MLST sequence types (STs), allelic profiles and toxin gene profiles of the 112 *C*. *difficile* culture positive clinical isolates.

ST (no. of isolates)	Clades	Allelic profile							Toxin genotype		
		*adk*	*atpA*	*dxr*	*glyA*	*recA*	*sodA*	*tpi*	*tcdA*	*tcdB*	*cdtA/cdtB *
ST-1 (3)	2	1	1	1	10	1	3	5	+	+	+/+
ST-2 (8)	1	1	1	2	1	5	3	1	+	+	-/-
ST-3 (12)	1	1	1	2	1	1	1	1	+	+	-/-
ST-3 (9)	1	1	1	2	1	1	1	1	-	-	-/-
ST-5 (3)	3	1	6	4	7	2	8	7	+	+	+/+
ST-8 (2)	1	1	1	2	6	1	5	1	+	+	-/-
ST-15 (5)	1	1	1	6	1	8	5	1	-	-	-/-
ST-26 (4)	1	1	1	6	1	4	3	4	-	-	-/-
ST-35 (6)	1	2	5	8	1	1	3	6	+	+	-/-
ST-37 (11)	4	3	7	3	8	6	9	11	-	+	-/-
ST-39 (8)	4	3	7	10	8	7	2	10	-	-	-/-
ST-42 (3)	1	1	1	2	1	1	7	1	+	+	-/-
ST-54 (15)	1	1	4	7	1	1	3	3	+	+	-/-
ST-55 (2)	1	1	1	6	6	1	12	12	+	+	-/-
ST-81 (9)	4	3	1	3	8	6	9	11	-	+	-/-
ST-91 (1)	1	1	1	6	6	1	6	1	+	+	-/-
ST-98 (1)	1	1	1	2	6	1	1	3	+	+	-/-
ST-100 (2)	1	1	1	6	19	2	24	1	-	-	-/-
ST-101 (1)	1	1	2	2	1	1	23	1	-	-	-/-
ST-117 (2)	1	1	1	7	1	2	5	1	-	-	-/-
ST-129 (2)	1	1	3	6	1	1	1	3	+	+	-/-
ST-286 (1)	1	1	1	2	3	1	5	3	+	+	-/-
ST-289 (1)	1	1	1	7	1	5	3	1	+	+	-/-
ST-320 (1)	4	11	7	10	43	6	20	15	-	-	-/-

## Discussion

CDI poses a great public health threat to health care facilities worldwide, with a significant rise in incidence, severity and mortality worldwide [16, 17]. Accurate and timely diagnosis of CDI is necessary for appropriate clinical management of the patients and infection control interventions [18, 19]. However, CDI is not widely recognized in Asia, including China. Although several *C*. *difficile* testing assays are now available worldwide, especially in North America and Europe, the VIDAS CDAB kit was the only commercial assay approved by China Food and Drug Administration as of December 2014. Furthermore, few laboratories routinely culture or carry out molecular-based detection of *C*. *difficile*. Therefore, the current laboratory diagnostic capacity for CDI detection in China, is inadequate to meet clinical demands, and thus there is an urgent need to introduce more practical *C*. *difficile* testing assays, including updating the current workflow, which would help in raising CDI awareness amongst clinicians [20, 21].

Several studies have demonstrated that GDH is a good screening test for *C*. *difficile* in fecal samples [22–25]. Our study is in agreement with these studies as GDH exhibited high sensitivity (100%) and favorable NPV (100%), compared with the culture based method. GDH testing can efficiently detect samples that are negative for CDI, with minimal hands-on time and cost. However, the detection of GDH simply indicates the presence of the organism and is not indicative of toxin production and thus cannot be used to diagnose CDI alone [11, 26].

The pathogenesis of CDI is mainly attributed to toxin A/B, which can be detected by VIDAS CDAB assay directly from fecal specimens. In the present study, the VIDAS CDAB assay showed good specificity (99.7%) and PPV (97.2%), but with low sensitivity (45%) and NPV (88.3%), compared with PCR-based toxin gene detection, which is in agreement to other studies [27]. Thus some CDI positive patients would be missed by using this test alone as a confirmatory test. PCR-based assays have high sensitivity and specificity [7, 8], but their costs exceed the affordability of many laboratories in developing countries.

To improve the sensitivity and cost-effectiveness of CDI diagnosis, we propose a GDH-based practical and efficient workflow, with combined testing methods in laboratory diagnosis of CDI. In this workflow, the VIDAS GDH assay is used as an initial screening assay, followed by detection of toxin production by VIDAS CDAB assay, and any discordant results between GDH and CDAB assays are confirmed by a third method such as molecular detection of toxin genes, toxigenic culture or cell culture cytotoxicity neutralization assay. By applying this proposed GDH-based workflow in this study, the CDI laboratory diagnosis rate was notably improved from 8.2% to 19.2%, yet the increasing cost was kept at the minimum level. This provides a good balance between increasing the CDI detection capacity for most China diagnostic clinical labs, and not using PCR, which as of October 2015 was not a legally authorized test.

In order to have some insight into the molecular epidemiology of *C*. *difficile* isolates in a part of China, MLST and toxin genotyping were performed. Our study revealed that the majority (71.4%) of the 112 isolates was toxin gene positive, and six isolates possessed the binary toxin gene, which is associated with higher mortality and recurrence rates [28]. MLST allows an easier inter-laboratory comparison of data and appears reasonably discriminatory for studying the transmission of *C*. *difficile* [15]. To date, this is the second study on MLST of *C*. *difficile* strains in China. Our findings showed that ST-3, ST-54 and ST-37 were the most common types at our hospital, which is in agreement to the first MLST study on 104 isolates in China [29], but different from other countries. For instance, in a tertiary care hospital in Spain, 34% of the isolates were ST-2, whilst in a teaching hospital in Japan, ST-17 (21.8%) was the most predominant ST [30, 31]. There was no obvious sign of outbreak as all the STs were distributed sporadically among different departments in our hospital.

This study has some limitations. First, all the specimens were collected from one single center and thus the efficiency and utility of the proposed workflow should be validated by more hospitals. Second, due to unavailability of commercial PCR kits in China for legal use, toxin gene detection was performed on *C*. *difficile* isolates after culture, instead of directly from fecal samples, which prolonged the turnaround time in the workflow.

The high positive rate of toxigenic strains in our study indicates that CDI may be an under-estimated problem in China. By introducing GDH as the screening test and properly applying the workflow, the diagnostic capacity and control of potential outbreaks of CDI may be improved significantly. Although GDH assay is not currently legalized for use in China, the licensing process is in the final stages, with indications of approval before end of 2015. Thus this proposed workflow is part of the preparatory work for best utilization of this test when it becomes available in China. The *C*. *difficile* strains in our study have been well preserved, and we are doing molecular epidemiology study of CDI. We hope our further study can provide more data and increase awareness and surveillance of CDI in China.
